# 

**Published:** 1894-05-12

**Authors:** 


CONTENTS.

Mr. Gladstone on Medicine - - - - - - - - 111
Around the Hospitals - - - - - - - - - 112
Artificial Respiration in Chloroform Collapse. By Sir Benjamin Ward Richardson, M.D., F.R.S. - - - - 113
Modern Medico-Psychology and Psychiatry.—V. - - 114
The Memorial to Sir Andrew Clark - - - - - 115
Annotations.—Opium or Alcohol?—Preventive Family Practice—Judges and Justice: Anderson v. Cook - - - - - 117
The Institutional Workshop—
Hospital Administration—Birmingham Hospital Saturday Fund - - - - - - - - - - 127
Hospital Festivals and Meetings - - - - - - - 129
Notes and News - - - - - - - - - - 118
Medical Progress and Hospital Clinics—
Congenital Displacement of the Head of the Femur and its Treatment - - - - - - - - - - 119
Cysts and Fistula Arising in Connection with a Persistent Thyro-Glossal Duct - - - - - - - - - - 120
London Hospital—Treatment of Acute Iritis - - - - 120
Medical Progress—Pleurisy - - - - - - - 122
Surgery of the Lungs and Pleuræ - - - - - - 123
Sub-Diaphragmatic Abcess - - - - - - - 124
New Drugs and Praparations - - - - - - - 125
New Appliances and Things Medical - - - - - 125

EXTRA SUPPLEMENT.—THE NURSING MIRROR.

News from the Nursing World.—"The Hospital" Convalescent Fund—An Excellent Association—Mrs. Brewer's Afternoon—The Nurses' Hotel—The Living and the Dead—How Not to Act in Emergencies—Inefficient or Educated?—Of an Uncertain Age—A Nurse for Builth—The Proposed Convalescent Home—Who is the Best Judge?—Melbourne A Fancy Picture—Short Items - - - - - - - li
On General Nursing. XI.—Pneumonia (*continued*) - - liii
The Advantages and Privileges of a Trained District Nurse and the Duties of the District Towards Her. By Henry C. Burdett - - - - - - - - - - liv
Appointments - - - - - - - - - - - lv
Royal National Pension Fund for Nurses v. The Record Press (Limited) - - - - - - - - - lv
The Wardroper Memorial - - - - - - - - lv
Queen Victoria's Jubilee Institute for Nurses - - - lvi
Wants and Workers - - - - - - - - - lvi
Our Annual Leave. A Holiday in Sark - - - - - lvii
Technical Education Committees and Nursing - - lviii
Minor Appointments—The Muses' Looking-Glass - - lix
Notes and Queries—Maternity Work in U.S.A. - - lx
For Reading to the Sick - - - - - - - - lx

